# Which methodologies and methods should be used for psychosocial intervention research in dementia? Protocol for a stakeholder-driven, multi-method Delphi study

**DOI:** 10.1136/bmjopen-2026-122720

**Published:** 2026-09-02

**Authors:** Sara Laureen Bartels, Federica D’Andrea, Alice Burnand, Simone R De Bruin, Ilaria Chirico, Emma Elliott, Aisling Flynn, Lesley Garcia, Doris Gebhard, Melanie Handley, Niels Janssen, Patricia Masterson-Algar, Marine Markaryan, Martina Roes, Nathan Stephens, Lieve Van den Block, Esme Moniz-Cook, Maud Graff

**Affiliations:** 1 Department of Psychiatry and Neuropsychology and Alzheimer Centrum Limburg, Mental Health and Neuroscience Research Institute, Maastricht University, Maastricht, The Netherlands; 2 Department of Clinical Neuroscience, Karolinska Institutet, Stockholm, Sweden; 3 The Geller Institute of Ageing and Memory, School of Medicine and Biosciences, University of West London, London, UK; 4 Division Health and Social Care, Research Group Living Well with Dementia, Windesheim University of Applied Sciences, Zwolle, The Netherlands; 5 Department of Human Sciences for Education “Riccardo Massa”, University of Milan-Bicocca, Milan, Lombardy, Italy; 6 National Institute for Health and Care Research (NIHR) Applied Research Collaboration-Greater Manchester, Faculty of Biology, Medicine and Health, School of Health Sciences, The University of Manchester, Manchester, UK; 7 School for Allied Health and Exercise Science, Faculty of Health, Environment and Medical Sciences, Bournemouth University, Poole, UK; 8 School of Sociology and Social Policy, University of Nottingham, Nottingham, UK; 9 Department of Health and Sport Sciences, Technical University of Munich, Munchen, Germany; 10 Centre for Research in Public Health & Community Care, University of Hertfordshire, Hatfield, UK; 11 School of Health Sciences, Bangor University, Bangor, UK; 12 German Center for Neurodegenerative Diseases, Witten, Germany; 13 Faculty of Health, Department of Nursing Science, University of Witten/Herdecke, Witten, Germany; 14 The Association for Dementia Studies, University of Worcester, Worcester, UK; 15 Institute for Implementation Science in Health Care, University of Zurich, Zürich, Switzerland; 16 VUB-UGent End-of-Life Care Research Group, Department of Family Medicine and Chronic Care, Vrije Universiteit Brussel, Brussels, Belgium; 17 Faculty of Health Sciences, University of Hull, Hull, UK; 18 Medical School, University of Exeter, Exeter, UK; 19 Department of Rehabilitation, Radboudumc, Radboud University Medical Centre Nijmegen, Nijmegen, The Netherlands; 20 Department of IQ Health, Radboudumc Alzheimer Center, Radboud University Medical Center, Nijmegen, The Netherlands

**Keywords:** Dementia, Psychosocial Intervention, Methods, Delphi Technique, Implementation Science

## Abstract

**Abstract:**

**Introduction:**

Psychosocial interventions are essential to support people living with dementia and their carers. A consensus on what is most important for research on psychosocial intervention in dementia and how intervention studies should best be conducted is currently lacking. This protocol describes the research plan aimed at achieving consensus on (i) the relevance of core elements (CEs) for the development, feasibility testing/piloting, evaluation and/or implementation phases of psychosocial interventions in dementia and (ii) methodologies (eg, design) and methods most suitable to address CEs per phase.

**Methods and analysis:**

This study was co-designed with a multi-stakeholder advisory group and a multi-disciplinary INTERDEM (psychosocial INTERventions in DEMentia) Methodology Taskforce steering committee. It will involve a multi-phase modified Delphi design, including surveys and group discussions with stakeholders, namely people living with dementia, (informal/unpaid/family) carers, health and social care professionals, policy makers, representatives from insurance companies and psychosocial researchers. A series of iterative ‘rounds’ will be conducted. In round 1 (Phase 1: ‘identification’), stakeholders will be asked to complete an online survey rating the importance of CEs from the UK Medical Research Council (MRC) Framework, namely (i) consider context; (ii) develop, refine and (retest) programme theory; (iii) engage stakeholders; (iv) identify key uncertainties; (v) refine interventions and (vi) economic considerations per phase; propose relevant additional CEs and list methodologies/methods that most suitably address CEs. These ratings will be further explored through online discussion rounds (Phase 2: ‘elaboration’). In round 2 (Phase 3: ‘consensus’), participants will be asked to rate the importance of CEs again (ie, new CEs and where no consensus was reached in Phase 1) and the usefulness of methodologies/methods to address CEs. Outcomes will be discussed with the advisory group and steering committee (Phase 4: ‘validation’). This process (Phases 3 and 4) will be repeated until a consensus on CEs and methodologies/methods is achieved.

**Ethics and dissemination:**

Ethical approval was received at Maastricht University (FHML-REC/2025/078) and the University of West London (UWL/REC/SBS-01195). Participants will sign informed consent prior to study participation. Results will be disseminated through a peer-reviewed publication, seminars, webinars, conferences, postgraduate dementia programmes, blogs, commissioner briefings and social media.

**Study registration:**

Open Science Framework (https://doi.org/10.17605/OSF.IO/DQRFA).

**STRENGTHS AND LIMITATIONS OF THIS STUDY**

- People with lived experience of dementia and healthcare professionals actively contribute through an advisory group to refine the methodological approach and research process.
- Multiple stakeholder groups will be recruited to achieve wide-ranging consensus.
- Survey ratings will be complemented with qualitative discussion rounds.
- Materials and data collection are limited to the English language.

## Introduction

 Across disease trajectories and aetiologies of dementia, psychosocial interventions are essential to support people living with dementia and their carers (eg,^1–3^). Psychosocial interventions, also known as non-pharmacological interventions, include activities, techniques, strategies, training, programmes and technology involving sensory, cognitive, behavioural, emotional, educational, social and/or environmental components targeting a range of outcomes, groups, settings and organisational levels.^4 5^ Examples of psychosocial interventions in dementia include art therapy,^6^ cognitive training,^7^ psychotherapy,^8^ aroma or touch therapy,^9 10^ occupational therapy^11^ and many more.^12^ These psychosocial interventions provide a holistic approach to improve outcomes such as quality of life, daily functioning, social health, mood and/or well-being across the trajectory for people with dementia^2 3^ and their carers.^13 14^

Research on psychosocial interventions in dementia can only have a sustainable and substantial societal impact if research questions are relevant and methodologies and methods are feasible, suitable and acceptable while also being heuristic to address a specific research question.^15^ The much-needed development, feasibility testing/piloting, evaluation and implementation of personal and socially relevant psychosocial interventions remain limited. Relevance ensures that psychosocial interventions are meaningful for the target groups, meet needs, preferences and capabilities, add value to the individual, organisation or society and are effective, sustainable and acceptable when adopted in the real world. The UK Medical Research Council (MRC) published a widely used framework for developing, feasibility testing/piloting, evaluating and implementing complex interventions,^16^ such as psychosocial interventions in dementia. As the authors highlight, any intervention that involves human behaviours, agency and social relationships (eg, interaction between someone delivering and receiving an intervention) is never simple, and even seemingly ‘simpler’ interventions have varying effects in different contexts.^17^ This definition of complexity highlights that psychosocial interventions in dementia are usually complex in nature, and dementia researchers can benefit from the MRC framework’s recommendations. Specifically, the MRC framework outlines a common set of core elements (CEs) across all phases, including: (i) consider context; (ii) develop, refine and (retest) programme theory; (iii) engage stakeholders; (iv) identify key uncertainties; (v) refine interventions and (vi) economic considerations.^16^ However, the MRC framework does not provide detailed guidance on how to address these CEs, nor is the framework specific to dementia research.

To our knowledge, the extent to which the MRC framework CEs are perceived as important by different dementia stakeholders, such as psychosocial researchers, health and social care professionals, policy makers, representatives from insurance companies and people with lived experiences of dementia, has not been explored. Furthermore, other factors perceived by these stakeholders as important when developing, feasibility testing/piloting, evaluating or implementing psychosocial interventions in dementia are unknown. In the blueprint for dementia research, the WHO highlights methodological issues, specifically a lack of appropriate methodologies that reflect priorities of people affected by dementia suitable for different cultural, regional and language contexts.^18^ As such, the WHO’s call for action emphasises the need for (i) representative research methodology and methods, including applying designs, recruitment and data collection approaches equitable for participation of underrepresented populations and research in environments as close to real-life settings as possible and (ii) harmonised methods and frameworks by, for instance, developing and disseminating health service research methods, harmonisation of frameworks for contextualised, inclusive research design and measurement and the promotion of research on implementation design and evaluation of interventions.^18^

Flexible and innovative methodologies and methods are warranted when conducting research with people living with dementia.^19^ Traditional methodologies (including designs) and methods, such as interviews, focus groups and randomised control trials, have been criticised as inadequate for understanding the complexity of dementia due to their reliance on verbal means of expression and abstraction alongside an omission of the complexity of healthcare contexts.^5^ Thus, a consensus on CEs, methodologies and methods for psychosocial interventions in dementia is needed. This consensus should also incorporate input from relevant stakeholders, such as people with lived experience of dementia, health and social care professionals, policy makers and representatives from insurance companies if these stakeholders are to have a tangible and meaningful impact on dementia policymaking and care practice.

### Study objectives

This study aims to elicit multi-stakeholder opinions and achieve consensus on (i) which methodological CEs are important for the development, feasibility testing/piloting, evaluation and implementation of psychosocial interventions in dementia and (ii) which methodologies and methods are most suitable to address these CEs in each phase (ie, development, feasibility testing/piloting, evaluation, implementation).

## Methods and analysis

### Study design

**Figure 1:**
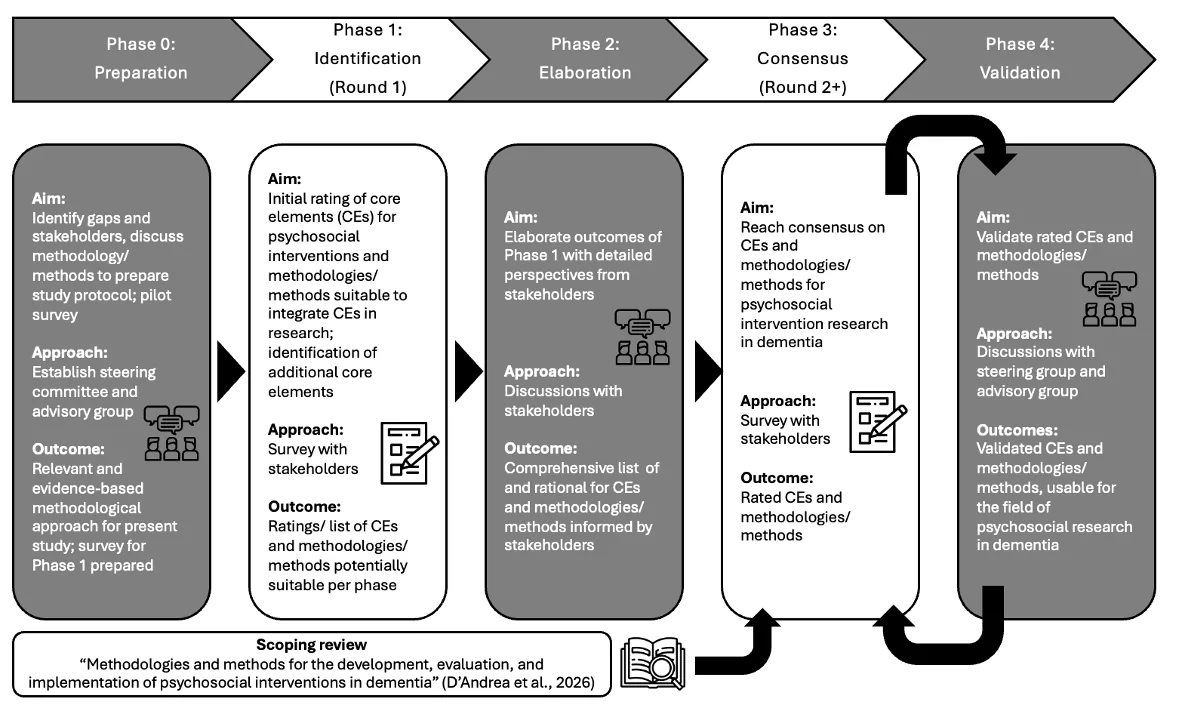
Study design. CEs, core elements.

A multi-phase study will be conducted using a stakeholder-driven modified Delphi consensus design approach by surveying a panel of professionals and lived experts to arrive at a group opinion or decision.^20^ The methodological details include innovative components, especially the involvement of people with lived experience of dementia and healthcare professionals in the design of the study as well as the final consensus and may inspire future dementia researchers: The present Delphi approach was co-designed in collaboration with a multi-stakeholder advisory group and a multi-disciplinary INTERDEM Methodology Taskforce steering committee as part of the METHODEM (METHOdological consensus for complex intervention in DEMentia) project.^4 5^ To make effective decisions in situations in which there is contradictory or insufficient information, Delphi techniques for reaching consensus are recommended.^21^ This iterative procedure includes a series of sequential surveys (‘rounds’) intertwined with additional stakeholder discussions. In round 1, stakeholders (as participants) will be asked to complete an online survey rating the importance of CEs from the UK MRC Framework, namely (i) consider context; (ii) develop, refine and (retest) programme theory; (iii) engage stakeholders; (iv) identify key uncertainties; (v) refine interventions and (vi) economic considerations per phase (ie, development, feasibility testing/piloting, evaluation, implementation)^16^; propose relevant additional CEs and list methodologies/methods that best address CEs. These ratings will be further explored through online discussion rounds. In round 2, participants will be asked to rate the importance of CEs again (ie, new CEs and where no consensus was reached in Phase 1) and the usefulness of methodologies/methods to address CEs. Outcomes of this round will be discussed with the advisory group and steering committee. This process of online surveys and discussions will be repeated until a consensus on CEs and methods/methodologies is achieved. Thus, the study will follow five phases, including ‘Phase 0: Preparation’, ‘Phase 1: Identification’, ‘Phase 2: Elaboration’, ‘Phase 3: Consensus’ and ‘Phase 4: Validation’. Moreover, findings from an ongoing scoping review focused on methodologies and methods to develop, evaluate and implement psychosocial interventions in dementia^4^ will feed into Phase 3. Phases 3 and 4 may be repeated if consensus among stakeholders is not reached. The design of the study is illustrated in figure 1 and further elaborated below. The planning of this study was also informed by the Guidance on Conducting and Reporting Delphi Studies (CREDES).^22^

### Phase 0: preparation

Phase 0 aims at identifying stakeholder groups, describing gaps in the field, defining research aims and questions and discussing the methodology and methods applied in the present study. Methodology is defined as the research strategies that determine how to conduct the study, including theoretical framework/paradigms, research approach and design, while methods refer to data collection and analysis.^4 23^ In the preparation phase, a steering committee and advisory group were established and involved in the study design.

#### Steering committee

The steering committee is composed of 21 multi-disciplinary researchers with backgrounds in (neuro-)psychology, sociology, occupational therapy, nursing, health sciences, implementation science and health promotion researchers, including the authors, working in the field of dementia research and care. All members of the committee are members of the Methodology Taskforce of INTERDEM, a pan-European multidisciplinary network focused on early, timely and quality psychosocial interventions in dementia to enhance practice, policy and quality of life for people with dementia and carers across Europe, ensuring that people with dementia and carers are involved in research activities (www.interdem.org). The role of the INTERDEM Methodology Taskforce, co-led by SLB, FD’A and MG, is to support the planning and executing of this study, data analysis and dissemination of findings. Lead researchers received some funding for this study (ARCOM-24-384 1250087) but the steering committee did not receive any financial incentives.

#### Advisory group

An advisory group was formed with the purpose of guiding the research and ensuring active involvement of key stakeholders in all stages of the research process, as recommended for other health conditions.^24–26^ This group is comprised of up to 12 members, including 8 people living with dementia, 2 carers and 2 healthcare professionals (ie, occupational therapist, nurse). Representatives from commissioners of health and social care, insurance companies and policy makers were also approached but could not be recruited for Phase 0. The role of the advisory group is to critically reflect, advise on and support the study planning, data analysis and dissemination with its own expertise. Advisory group members living with dementia and carers are reimbursed for their time aligning with the NIHR guidance for researchers and professionals involving people in research.^27^

#### Identification of stakeholder groups

Throughout the study, six stakeholder groups were identified to be included as participants. Specific in- and exclusion criteria per stakeholder groups are outlined below:

1. *People living with dementia*: People diagnosed with any type of dementia.
2. *Carers (informal/unpaid/family*): People in a close personal relationship to a person with dementia, such as partners, children, siblings, other relatives or close friends, who are currently providing informal care and support or have done so within the past 2 years, often unpaid.
3. *Health and social care professionals*: People currently or recently employed within the past 2 years by a public or private institution that provides support in a health or social care setting for people living with dementia and/or carers, including both care and support providers as well as managers.
4. *Insurance companies:* People assessing and potentially funding or reimbursing costs of psychosocial interventions to health and social care organisations or private individuals.
5. *Policy makers*: People shaping or informing national, regional or local dementia policies or strategies or commissioning dementia service provision.
6. *Psychosocial dementia researchers*: People currently or recently involved within the past 2 years in undertaking or collaborating on research into psychosocial interventions for dementia.

### Phase 1: identification

The aim of Phase 1 is to identify (i) CEs for the development, feasibility testing/piloting, evaluation and implementation of psychosocial interventions in dementia and (ii) methodologies and methods suitable to address these CEs in research per phase as indicated by researchers and other stakeholders. The six MRC CEs will serve as a starting point, and participants will be asked in a survey to rate these CEs regarding their importance (per phase), suggest additional CEs and indicate methodologies and methods they have used, are currently using or want to use prospectively to address them.

#### Study sample

While there is no consensus on the sample size needed for Delphi studies,^28^ other Delphi and consensus studies involving people with lived experiences of a health condition, carers and/or healthcare professionals aimed for or included between 25 and 240 participants, often with no or limited justification.^29–33^ In the present study, we aim for approximately 200 participants, including 60–100 researchers and 10–20 participants for other stakeholder groups. This sample size is chosen to reflect different perspectives as a form of saturation, a principle used in qualitative studies,^34^ while also considering feasibility of recruitment and time availability.

For the discussion rounds, online interactions will include between two and five participants (based on stakeholder group, capacity and preference) to facilitate easy interactions. For in-person conversations, group sizes are anticipated to range between six and eight participants (size will depend on the stakeholder group, participant availability and preferences). In these specific discussion rounds, sample size is informed by the concept of information power, where a small number of participants hold relevant expertise.^35^ If needed, individual conversations with a member of the research team will be arranged to address participants’ needs. The groups may be mixed, consisting of different stakeholders where feasible.

#### Inclusion and exclusion of participants

Inclusion and exclusion criteria of the six stakeholder groups are presented in table 1. No formal eligibility check will be performed; instead, participants confirm their eligibility based on self-report in the online survey for each phase. Participants will be asked to read a study information sheet tailored to the stakeholder group and sign an electronic informed consent (e-Consent) by selecting a response from a drop-down menu (yes/no). Participants may also consent (yes/no) to be contacted for follow-up survey rounds and discussion rounds. It will be reiterated that participation is voluntary.

**Table 1:** Inclusion and exclusion criteria

Stakeholder group	Inclusion criteria	Exclusion criteria
People with dementia	>18 years old, AND Diagnosed with dementia (based on self-report), AND Able to communicate in English, AND Have access to a computer to complete an online survey.*	Diagnosed with mild cognitive impairment or another neurological condition
Carers	>18 years old, AND Currently are or previously (past 2 years) were a carer for a person with dementia (based on self-report), including often unpaid† spouses, children, other members or close friends (note: the person with dementia may be living at home or in a care home), AND Able to communicate in English, AND Have access to a computer to complete an online survey.	Being a carer of a person with other care needs and conditions
Health and social care professionals	>18 years old, AND Able to communicate in English, AND Have access to a computer to complete an online survey, AND Are currently or were previously (past 2 years) responsible for providing care and treatment to people with dementia and/or unpaid carers, including (but not limited to) general practitioners, medical doctors, (clinical/health/neuro-) psychologists, nurses, occupational therapists, case managers, social workers, allied health professionals and professionals who run community-based programmes.	Care and treatment focused only on people with other conditions
Insurance companies‡	>18 years old, AND Able to communicate in English, AND Have access to a computer to complete an online survey, AND Work currently or previously (past 2 years) at a regional, national or international (eg, European) level for a health/social care insurance company to reimburse or otherwise support psychosocial interventions for dementia care.	Health insurance focus only on other populations/conditions
Policy makers	>18 years old, AND Able communicate in English, AND Have access to a computer to complete an online survey, AND Are or were (past 2 years) actively involved in dementia-related policy work at a regional, national or international (eg, European) level, including governmental departments, health and social organisation, regulatory and advisory bodies and third-sector organisations.	Policy work focuses only on other populations
Psychosocial dementia researchers	>18 years old, AND Able to communicate in English, AND Have access to a computer to complete an online survey, AND Currently or previously (past 2 years) working part- or full-time for an institute, university or other research setting anywhere in the world, AND Executing or collaborating on research on the development, feasibility testing/piloting, evaluation or implementation of psychosocial interventions targeting people with dementia at any stage, type or setting and/or their unpaid carers.	Primary research focus on other topics, such as dementia prevention, biomedical or pharmaceutical interventions/treatment or other neurological conditions (eg, mild cognitive impairment)

* Assistance for completing the survey was offered via the phone or a video call.

† Following the feedback from a carer (ie, partner of a person living with dementia) who received a small financial compensation for assisting their spouse with dementia, the term ‘carer’ was used instead of ‘unpaid/ informal/ family carer’ as it was perceived as the most neutral wording.

‡ In the UK, this might also include commissioners of health and social care.

For people living with dementia to participate in this study, they need to have the capacity to make an informed choice. As the members of the advisory group are all UK-based, the Mental Capacity Act (MCA)^36^ was used to define capacity, stating that a person will be assumed to have capacity unless it is established otherwise. This perspective on capacity and informed consent is also in line with German consent guidelines.^37^ The advisory group also offers input on the consent procedure, following recommendations on public involvement from the European Working Group of People with Dementia.^38^

Thus, people living with dementia will be flexibly supported in the consent process, placing them at the centre of the consent procedure and facilitating autonomy and decision-making.^39^ For the online survey, it will be explained (in text and verbally, where requested) that people living with dementia may involve their carer to assist in understanding the study requirements, consent procedure and answering the survey questions. They may also request support from a researcher (SLB) who will assist via the phone.

For the discussion rounds in Phases 2 and 4, the researcher team, consisting of experienced dementia researchers, will be in contact with participants via email/phone to assess the following: 1) the capacity to understand the information relevant to the decision to be made, 2) retain the information sufficiently to engage in direct conversation, 3) use or weigh the information to arrive at a choice/decision and (4) ability to communicate a decision. In line with the MCA Code of Practice,^36^ if an individual is unable to meet any of the first three assessment criteria, they will be regarded as lacking capacity for that specific decision. The fourth criterion is relevant only when a person has trouble communicating their decision.

#### Recruitment strategies

Study information and an invitation to participate will be shared online through various channels. Specifically, the following strategies will be used to reach a wide and international audience:

- Social media posts on LinkedIn, posted by lead researchers and from METHODEM project page with a request to reshare.
- Emails and messaging within existing networks, such as INTERDEM Newsflash/Taskforces, Walking the Talk for Dementia community and departments of executing researchers.
- Posts on pages of the Alzheimer’s Association International Society to Advance Alzheimer’s Research and Treatment Professional Interest Areas: ‘Partnering with Research Participants’, ‘to Elevate Early Career Researchers’ and ‘Nonpharmacological interventions’.
- Tailored emails and social media messages to specific individuals or groups, identified through social media searches and snowball sampling.

#### Survey

The survey will be conducted online using REDCap securely stored on the servers of Maastricht University (the Netherlands). Three tailored surveys will be created for Phase 1: a version for psychosocial dementia researchers, a survey version for people with lived experience (ie, people living with dementia and carers) and a version for all other stakeholders. The researcher survey was co-created with the steering committee: a draft version was shared via email, and the steering committee members provided suggestions, for instance, to refine the wording (ie, use ‘I’ statements instead of ‘it’ statements) and rating scale (ie, use a 5-point scale). Similarly, the other two surveys were co-created with the advisory group. For the survey for people living with dementia and carers specifically, the drafted version was circulated via email, and feedback was discussed during a 60-min online meeting with six advisory group members living with dementia. Broad questions such as “what did you think of the survey?” and “what should be changed about the survey and why?” were asked to facilitate discussion. The remaining advisory group members provided feedback via email or attended individual online meetings. Key insights from the advisory group members were simplifying the wording, increasing the font size and reducing the amount of text and length of the survey.

All surveys will include a section on sociodemographic information (ie, age, sex, gender identity, country of residence). Additionally, people living with dementia will be asked about the type of dementia and number of years living with dementia (since diagnosis); carers will be asked about the relationship with the person living with dementia, type of dementia, stage of dementia (ie, mild, mild-moderate, moderate, moderate-advanced, advanced) and the number of years of providing care. Health and social care professionals, policy makers and representatives from insurance companies will be asked to describe their professional role, the number of years working in this role in dementia care and country of employment. Researchers will be asked about the country of employment, position and academic background.

**Figure 2:**
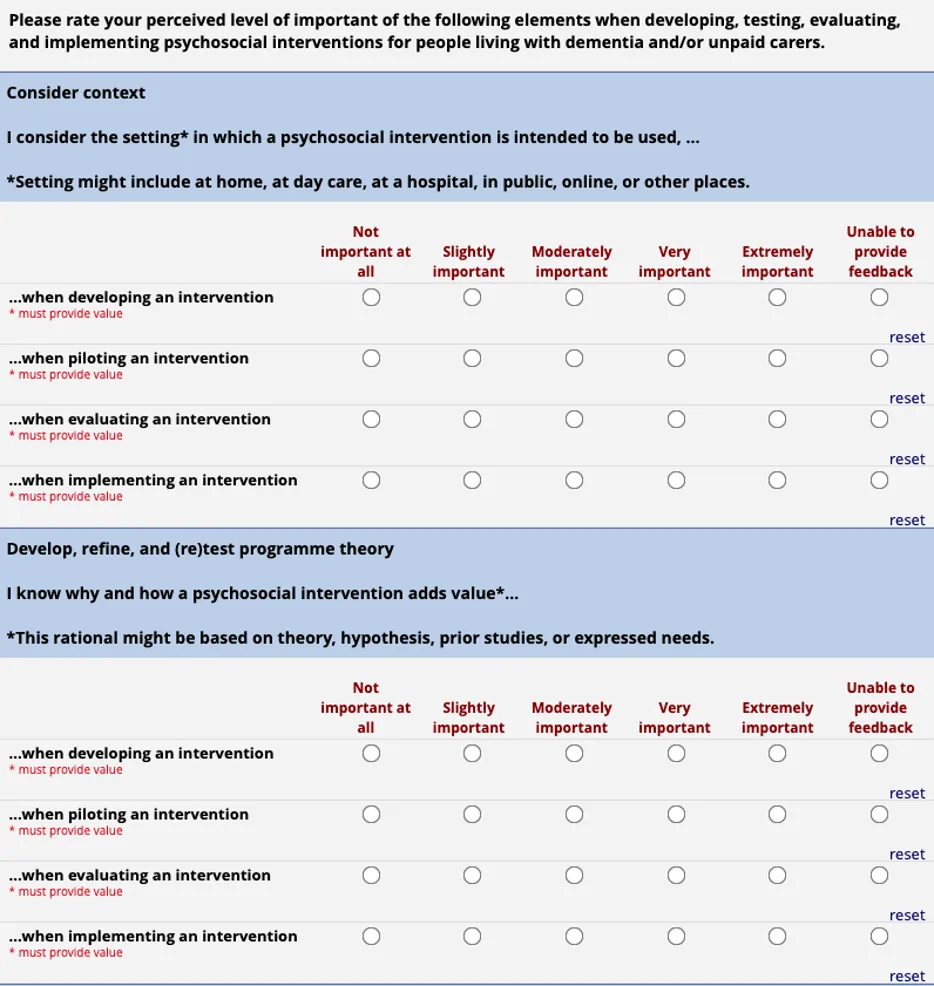
Example—survey version for researchers.

**Figure 3:**
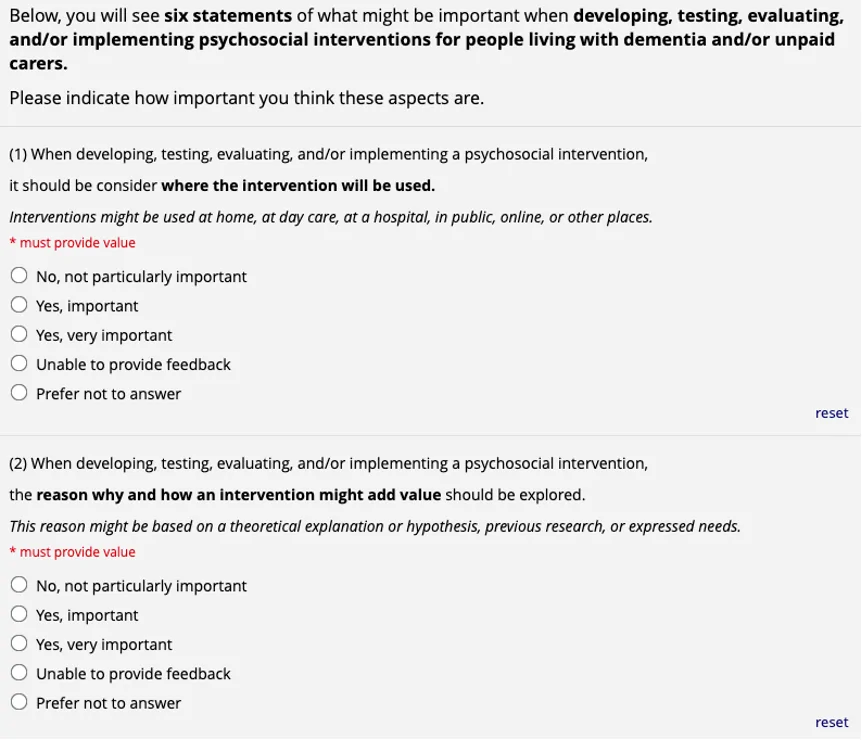
Example—survey version for other stakeholders.

Following, participants will be asked to complete the survey, rating the importance of the MRC CEs. In Phase 1, the survey version for researchers includes a rating of each CE per phase on a 5-point scale (ranging from ‘Not important at all’ to ‘Extremely important’) (figure 2, online supplemental appendix 1), while the version for the other stakeholder groups includes an overall rating of each CE on a 3-point scale (ranging from ‘Yes, very important’ to ‘No, not particularly important’) (figure 3) (online supplemental appendix 2). The 3-point scale was selected following the modified Delphi approach used by,^40^ in order to reduce the cognitive demands for participants.

Researchers will also be asked how many years they have conducted research related to psychosocial interventions for people living with dementia and/or carers, in which phase(s) their research was/is situated (ie, development, feasibility testing/piloting, evaluation, implementation) and, keeping the MRC CEs in mind, which design and/or methods they would recommend when developing, feasibility testing/piloting, evaluating and/or implementing these interventions. All participants may propose additional elements to consider for psychosocial intervention research and provide feedback to the research team through open questions.

#### Analysis of survey responses

Descriptive statistics (frequency, percentage) will be used to summarise survey and demographic data, also considering group allocation. For each CE, the distribution of rating scores will be presented in a histogram and/or tables. Additional CEs suggested by participants will be reviewed and discussed with members of the steering committee and advisory group. Moreover, the mentioned methodologies and methods will be listed and may be inductively and deductively summarised in categories (eg, qualitative/quantitative methods, cross-sectional/longitudinal designs). All outcomes will be carried forward to Phase 2.

### Phase 2: elaboration

#### Discussions with stakeholders

Qualitative data will be used to elaborate survey responses with in-depth perspectives of stakeholders from the discussion rounds. Specifically, group discussions and individual interviews will be conducted. Participants may have joined Phase 1 or be new to the study. New participants will be asked to provide written informed consent and complete a short questionnaire online. Additionally, at the start of the group discussion rounds, verbal consent will be requested.

The conversations will follow a topic guide, including (i) a warm-up phase (ie, getting to know each other, exploring links to psychosocial interventions in dementia and experience with participation in or collaboration with research), (ii) the main conversation and (iii) a closing where key messages will be summarised and next steps outlined. The main conversation will centre around the survey findings and focus on how stakeholders relate to or interpret outcomes from their perspective, knowledge and experience. Specifically, one or more researchers will explain in lay language (rating of) CEs, methodologies and methods. Next to verbal explanations and questions, visual and object prompts may be used, such as descriptions of specific research projects using certain methods and methodologies, pictures or graphics illustrating engagement or displaying other study materials (eg, questionnaires, digital diaries). The use of a range of approaches is inspired by Hogger *et al*,^41^ and materials will be refined together with the steering committee and advisory group. All group discussions will be recorded and fieldnotes will be taken. Discussions will be transcribed using the transcription function in MS Teams and checked by a researcher for accuracy.

#### Analysis of qualitative data

A rapid approach combining a framework (for CEs) and inductive reasoning (for methodologies/methods) to processing the qualitative data of discussion rounds and interviews will be used, where one researcher (SLB) will create codes and themes and select quotations using ATLAS.ti. These codes and themes will be presented to and discussed with the core team (FD’A, MG) and steering committee for cross-checking and refinement.

### Phase 3: consensus

Participants who indicated their interest in taking part in follow-up surveys in Phase 1 will be contacted again for participation in Phase 3. This survey will be built with the steering committee and advisory group and include findings from Phases 1 and 2. Specifically, participants will receive a document with their own scores, group scores and qualitative themes. Participants will then be asked to rate the importance of CEs again (where no consensus was reached in round 1), potentially adjusting their response. Consensus is defined as follows^40^:

- Consensus in: >70% of participants score the item as ‘very important’ and <15% of participants score it as ‘not important’.
- Consensus out: >70% of participants score the item as ‘not important’ and <15% of participants score it as ‘very important’.
- No consensus: Any percentage of scoring not included in these two categories.

Moreover, participants will be asked to rate the suitability of specific methodologies and methods to address CEs. The list of methodologies and methods will also be informed by the METHODEM scoping review.^4^ While this review is being conducted in parallel to the Delphi study, data extraction has already resulted in a preliminary overview of methodologies and methods used to develop, test, evaluate and implement psychosocial interventions for dementia (Status August 2026). Specifically, this overview will be compared with methodologies and methods mentioned in Phase 1, discussed in Phase 2 and potentially added to the consensus items in Phase 3. A short description of methodologies and methods may be provided. All outcomes of Phase 3, including consensus in, consensus out and no consensus on CEs as well as methodologies and methods, will be qualitatively discussed in Phase 4.

### Phase 4: validation

The findings of Phase 3 will be presented to the steering committee and advisory group for discussion and validation of the ratings. Depending on the outcomes of Phase 3 and the input from these groups, additional survey rounds may be prepared and executed to reach a consensus. These survey rounds may be complemented by the overview of methodologies and methods resulting from the METHODEM scoping review.^4^

### Status of the study and reporting

Ethical approval was received (October 2025), and Phases 0 and 1 were completed in April 2026. Analysis of data collected in Phase 1 is ongoing, and Phase 2 is being prepared (August 2026) to take place in September 2026. Phases 3 and 4 are planned for October/November 2026.

The process and results of the Delphi study will be reported following the CREDES guidance^22^ and complemented with the ACCORD (ACcurate COnsensus Reporting Document) guideline^42^ to ensure transparency on how practical recommendations and potential adaptations to the MRC framework were made and should be interpreted.

### Patient and public involvement

An advisory group is involved in the refinement of the study design, execution (ie, reviewing materials) and interpretation (Phase 4: Validation) and might be involved in dissemination activities.

## Ethics and dissemination

Ethical approval was received at Maastricht University (FHML-REC/2025/078) and the University of West London (UWL/REC/SBS-01195). Participants will sign informed consent prior to study participation.

The process and findings of the present study will be transparently described in a scientific article and submitted for peer review. Moreover, findings will be disseminated at dementia and other relevant conferences, the annual INTERDEM business meeting, the INTERDEM news flash and integrated into at least one INTERDEM Academy masterclass, educating early- and mid-career researchers. Outcomes will also be shared via social media and sent to participants to share with their networks. For dissemination, both an academic and a lay-language summary will be created to increase access to the scientific insights for a wide audience. The target audience includes dementia researchers and those from other fields who work towards improving the lives of people with dementia and carers, from pre-diagnostic stages up to end of life and into bereavement. As such, this work will have a large impact across sectors and disciplines.

## Discussion

People living with dementia require complex care, and carers also require adequate support. Research into dementia care must thus be based on both sound evidence from health systems and robust clinical and intervention science.^18^ Psychosocial interventions are an essential pillar of dementia care and support.^43^ As such, this study aims at strengthening the evidence base underlying these interventions. The outcome of the Delphi study, namely a multi-stakeholder consensus on CEs and methodologies and methods, can thus be seen as a direct response to the WHO’s strategic goal 11: ‘*Tools and methodologies for interventions – Have high-quality tools and methodologies for the design, adaption, and evaluation of dementia care interventions that are applicable internationally and can be adapted locally’* (p. 42^18^).

The methodological approach of this Delphi study ensures relevance by involving multi-disciplinary researchers, people with lived experiences and health and social care professionals in the planning and execution of the study as well as interpretation of findings. It also aims to inspire researchers who are involved in developing, feasibility testing/piloting, evaluating and/or implementing psychosocial intervention research to make use of other, more appropriate or more innovative methods and methodologies. Moreover, adapting, for instance, the rating options to a 3-point scale as suggested by Harding *et al*,^40^ and piloting materials, such as the online survey, enhances feasibility and promotes a positive participant experience. The combination of quantitative ratings with qualitative insights also offers the possibility to reach a consensus, while diving deeper into the rationale. Finally, while the MRC framework and its CEs^16^ are widely used across research fields, disciplines and countries (eg,^44–47^), this study will highlight its relevance in the dementia field and might result in iterative refinements in response to identified methodological gaps. As such, the outcomes are expected to advance dementia and intervention research, inspiring researchers involved in developing, feasibility testing/piloting, evaluating and implementing psychosocial interventions.

### Anticipated challenges and limitations

In Phases 0 and 1, it became apparent that some stakeholder groups, namely policy makers and representatives from insurance companies, may be hard to reach. Snowball sampling and tailored messaging may facilitate establishing contact; yet, the recruitment reach will be limited, and the potentially low motivation of these stakeholders to join research might be challenging. As such, the final sample sizes across stakeholder groups may differ. Similarly, industry partners developing, feasibility testing/piloting, evaluating and selling psychosocial interventions may be a prospective stakeholder group to consider in future studies. In the present scope, the focus lay on interventions available through healthcare; thus, these stakeholders were not included. Finally, a limitation of this study is the inclusion criteria that participants need to be able to speak English to complete the survey or join discussion rounds. This focus is necessary as the resources, especially time, do not allow for translations and analysis in several languages. Nevertheless, this approach may create a bias towards highly educated individuals, especially for non-native speakers.

## Supplementary material

10.1136/bmjopen-2026-122720 online supplemental file 1

10.1136/bmjopen-2026-122720 online supplemental file 2
