# Supplementary material for: Which methodologies and methods should be used for psychosocial intervention research in dementia? Protocol for a stakeholder-driven, multi-method Delphi study
Source: BMJ Open. 2026 Sep 2;16(9):e122720. doi: 10.1136/bmjopen-2026-122720 (PMC13548282; doi:10.1136/bmjopen-2026-122720)
Supplement: Supplementary file 1 [file bmjopen-16-9-s001.pdf]

Researcher survey

Survey Queue

A A A

Thank you for signing informed consent!

Please start the main survey by **clicking the button 'BEGIN SURVEY'** below.

Please complete the following demographic questions.

Age

Sex

- ◯ Born male
- ◯ Born female
- ◯ Prefer not to answer

Gender

- ◯ Man
- ◯ Woman
- ◯ Self-describe
- ◯ Prefer not to answer

Country of residence

Country of employment

Position

- ◯ Research assistant
- ◯ PhD student
- ◯ Postdoctoral researcher
- ◯ Assistant Professor
- ◯ Associate Professor/ reader
- ◯ Lecturer
- ◯ Senior Lecturer

- ◯ Professor
- ◯ Other

Academic background(s)

- ☐ Psychology/ clinical psychology/ neuropsychology
- ☐ Nursing
- ☐ Occupational Therapy
- ☐ Medicine (e.g., neurology, psychiatry, gerontology)
- ☐ Behavioural sciences
- ☐ Epidemiology
- ☐ Data science
- ☐ Sociology
- ☐ Arts and design
- ☐ Physiotherapy
- ☐ Social care
- ☐ Health sciences
- ☐ Speech and language therapy
- ☐ Other

If you selected 'other' for 'Academic background', please add details here:

The objective of this study is to reach a **methodological consensus** in the field of psychosocial interventions in dementia. We are interested in your opinions on how to best develop, test, evaluate, and/or implement psychosocial interventions for people with dementia and/or unpaid carers, such as partners, family members, or close friends. Psychosocial interventions may include, for example:

- Psychological support to cope with challenges in everyday life
- Occupational therapy to promote engagement in meaningful activities
- Speech and language therapy to train speaking and communication
- Sensory stimulation such as massages or touch therapy
- Physical activities, such as walking, exercising, or yoga
- Group and social activities to feel connected and understood, including dementia cafes and support groups
- Education to know what dementia is and how to live with it
- Cognitive stimulation therapy or rehabilitation to improve memory
- Environmental designs or adaptations such as the use of certain lights, textiles, or objects to offer comfort

Many more psychosocial interventions are available that can **support the quality of life** of the person with dementia and/ or unpaid carer. Quality of life could include the person's well-being, daily functioning,

engagement in activities, mood, relationship quality, or any other important aspect of their life that is relevant to them.

Note: Psychosocial interventions do not include medications or pharmaceuticals, therapies that only improve physical outcomes (e.g., reduced heart rate or weight loss), or programs that focus on prevention.

Aim 1: In this study, we want to understand what is most important when psychosocial interventions are being developed, tested, evaluated, or integrated into practice.

Key definitions of research phases:

- **Developing** meaning designing or planning an intervention, and it might include creating a new intervention or adapting an existing intervention to specific people or environments.
- **Feasibility/piloting**: Testing means checking 'Can this be done?'; for instance, if people can and want to use, deliver, or support the intervention.
- **Evaluating** means checking what kind of benefits or impact the intervention has on the person or the system in which it is used.
- **Implementation** means integrating an intervention into practice, including specific actions or efforts taken to increase the chance that the intervention is being used long-term and has its intended impact.

Specifically, we want to understand how important researchers find the proposed 'six core elements' of the Medical Research Framework (Skivington et al., 2021), per phase, for psychosocial interventions in dementia:

- **Consider context**
- **Develop, refine, and (re)test program theory**
- **Engage stakeholders**
- **Identify key uncertainties**
- **Refine intervention**
- **Economic considerations**

Please rate your perceived level of important of the following elements when developing, testing, evaluating, and implementing psychosocial interventions for people living with dementia and/or unpaid carers.

Consider context

I consider the setting* in which a psychosocial intervention is intended to be used, ...

***Setting might include at home, at day care, at a hospital, in public, online, or other places.**

	Not important at all	Slightly important	Moderately important	Very important	Extremely important	Unable to provide feedback
**...when developing an intervention** * must provide value	○	○	○	○	○	○
**...when piloting an intervention** * must provide value	○	○	○	○	○	○
**...when evaluating an intervention** * must provide value	○	○	○	○	○	○
**...when implementing an intervention** * must provide value	○	○	○	○	○	○

Develop, refine, and (re)test programme theory

I know why and how a psychosocial intervention adds value*...

***This rational might be based on theory, hypothesis, prior studies, or expressed needs.**

	Not important at all	Slightly important	Moderately important	Very important	Extremely important	Unable to provide feedback
...when developing an intervention<br>* must provide value	○	○	○	○	○	○
...when piloting an intervention<br>* must provide value	○	○	○	○	○	○
...when evaluating an intervention<br>* must provide value	○	○	○	○	○	○
...when implementing an intervention<br>* must provide value	○	○	○	○	○	○

Engage stakeholders

I engage people who will be in contact* with the psychosocial intervention ...

*** In contact refers to**
People who receive the intervention such as people living with dementia or unpaid carers
People who deliver the intervention such as health and social care professionals
People who support the intervention such as healthcare mangers, insurance companies, or policy makers
People who do research on the intervention
Other people somehow relevant to the intervention

	Not important at all	Slightly important	Moderately important	Very important	Extremely important	Unable to provide feedback
...when developing an intervention<br>* must provide value	○	○	○	○	○	○
...when piloting an intervention<br>* must provide value	○	○	○	○	○	○
...when evaluating an intervention<br>* must provide value	○	○	○	○	○	○
...when implementing an intervention<br>* must provide value	○	○	○	○	○	○

Economic considerations

I consider psychosocial intervention costs and who funds it...

	Not important at all	Slightly important	Moderately important	Very important	Extremely important	Unable to provide feedback
...when developing an intervention<br>* must provide value	○	○	○	○	○	○

...when piloting an intervention<br>* must provide value	◯	◯	◯	◯	◯	◯
...when evaluating an intervention<br>* must provide value	◯	◯	◯	◯	◯	◯
...when implementing an intervention<br>* must provide value	◯	◯	◯	◯	◯	◯

Refine intervention

I refine* a psychosocial intervention...

*** Refinement may include any kind of changes or adaptations to the intervention e.g., design, set-up, materials, delivery etc. necessary to improve it or its impact.**

	Not important at all	Slightly important	Moderately important	Very important	Extremely important	Unable to provide feedback
...when developing an intervention<br>* must provide value	◯	◯	◯	◯	◯	◯
...when piloting an intervention<br>* must provide value	◯	◯	◯	◯	◯	◯
...when evaluating an intervention<br>* must provide value	◯	◯	◯	◯	◯	◯
...when implementing an intervention<br>* must provide value	◯	◯	◯	◯	◯	◯

Identify key uncertainties

I explore (other) key uncertainties*...

*** Factors that are uncertain might be related to the research process, such as how to recruit participants or what the benefits of the intervention could be but also connected to the intervention itself. For instance, it might generally be important to know if health or social care professionals have the competences, knowledge, and skills to deliver the intervention, or if people with dementia/ unpaid carers would accept and like it.**

	Not important at all	Slightly important	Moderately important	Very important	Extremely important	Unable to provide feedback
...when developing a psychosocial intervention<br>* must provide value	◯	◯	◯	◯	◯	◯
...when piloting a psychosocial intervention<br>* must provide value	◯	◯	◯	◯	◯	◯
...when evaluating a psychosocial intervention<br>* must provide value	◯	◯	◯	◯	◯	◯
...when implementing a psychosocial intervention<br>* must provide value	◯	◯	◯	◯	◯	◯

Please indicate any other important **considerations when developing** a psychosocial intervention for people living with dementia and/or unpaid carers.

Please indicate any other important **considerations when piloting** a psychosocial intervention for people living with dementia and/or unpaid carers.

Please indicate any other important **considerations when evaluating** a psychosocial intervention for people living with dementia and/or unpaid carers.

Please indicate any other important **considerations when implementing** a psychosocial intervention for people living with dementia and/or unpaid carers.

Aim 2: We also want to understand how researchers can best conduct research related to psychosocial interventions for people with dementia and/or unpaid carers. We are therefore interested in suggestions from researchers on which **designs and methods** should be used.

Designs refer to the 'blue-print' of the research and might include, for example:

- Single-arm trials
- Controlled trials
- Observational
- Longitudinal
- Cross-sectional
- Participatory
- Experimental
- Etc.

Methods refer to the form of data (collection, analysis, and interpretation) and might include, for example:

- Focus groups
- Interviews
- Questionnaires/ surveys
- Neuropsychological tests
- Experience Sampling
- Passive sensing
- Photo-elicitation/ photo-voice
- Observations
- Etc.

In this survey, we are interested in your general suggestions for designs and methods. In the subsequent survey rounds, we will reach a consensus on which of these proposed designs and methods consider the core elements best.

The questions below have been tailored to your level of expertise (e.g., development, piloting, evaluation, and/or implementation) and research focus (e.g., interventions for people living with dementia and/or unpaid carers).

My research focuses/ focused on psychosocial interventions **for people living with dementia.**

* must provide value

○ yes
○ no

My research focuses/ focused on psychosocial interventions **for unpaid carers** of people living with dementia.

* must provide value

○ yes
○ no

Please add **any further reflections or feedback** you might want to share with the researchers on this topic:

Submit

Save & Return Later

Powered by REDCap - Cookie policy
