# Supplementary material for: Which methodologies and methods should be used for psychosocial intervention research in dementia? Protocol for a stakeholder-driven, multi-method Delphi study
Source: BMJ Open. 2026 Sep 2;16(9):e122720. doi: 10.1136/bmjopen-2026-122720 (PMC13548282; doi:10.1136/bmjopen-2026-122720)
Supplement: Supplementary file 2 [file bmjopen-16-9-s002.pdf]

Survey Queue

Survey for health/ social care professionals, policy makers, and representative from insurance companies

AAA

Thank you for signing informed consent!

Please start the main survey by **clicking the button 'BEGIN SURVEY'** below.

Number of years of working in this role

Age

Sex

* must provide value

- Born male
- Born female
- Prefer not to answer

Gender

* must provide value

- Woman
- Man
- Self-describe
- Prefer not to answer

Country of residence

Country of employment

We are interested in your opinion on non-pharmacological support, also known as '**psychosocial interventions**', for people living with dementia and/or unpaid carers, which may include, for example:

- Talk therapy to support coping with challenges in everyday life
- Speech and language therapy to help with speaking and understanding others
- Sensory stimulation, like massages or touch therapy
- Exercise, such as walking or yoga
- Group activities to feel connected and understood, including dementia cafés and support groups
- Education to know what dementia is and how to live with it
- Cognitive therapy to improve memory
- Environmental changes such as the use of lights, objects, or safe layout to help the person feel comfortable in their space

These interventions all aim to improve quality of life and **do not involve talking medication**. Quality of life could include the person's well-being, daily functioning, involvement in activity, mood, relationships, or any other aspect of their life that is relevant to them.

In this study, we want to understand **what is important** when:

1. Developing, meaning creating a psychosocial intervention
2. Testing, meaning checking if a psychosocial intervention is easy to use, acceptable, practical, and feasible
3. Evaluating, meaning examining a psychosocial intervention and checking it if works
4. Implementing, meaning putting a psychosocial intervention into real-life and everyday settings

Please answer the following questions:

Have you previously been **involved or participated in a psychosocial intervention research project**?

Specifically, a project focused on a psychosocial intervention for people with dementia and/or unpaid carers

* must provide value

- [ ] No, I have never participated in an intervention research project
- [ ] Yes, the intervention was being develop or (co-)created
- [ ] Yes, the intervention was being tested to see it it works
- [ ] Yes, the intervention was evaluated, to check if or how it works, or not
- [ ] Yes, the intervention was being put into real life practice (implementation)
- [ ] I don't know
- [ ] Prefer not to answer

If you selected 'I don't know', please provide details below:

Below, you will see **six statements on aspects to consider when developing, testing, evaluating, and/or implementing psychosocial interventions for people with dementia and/or unpaid carers.**

Please indicate how important you think these aspects are.

(1) When developing, testing, evaluating, and/or implementing a psychosocial intervention,

it should be considered **where the intervention will be used.**

Interventions might be used at home, at day care, at a hospital, in public, online, or other places.

* must provide value

- ○ No, not particularly important
- ○ Yes, important
- ○ Yes, very important
- ○ Unable to provide feedback
- ○ Prefer not to answer

(2) When developing, testing, evaluating, and/or implementing a psychosocial intervention,

the **reason why and how an intervention might add value** should be explored.

This reason might be based on a theoretical explanation or hypothesis, previous research, or expressed needs.

* must provide value

- No, not particularly important
- Yes, important
- Yes, very important
- Unable to provide feedback
- Prefer not to answer

(3) When developing, testing, evaluating, and/or implementing a psychosocial intervention,

people who will receive, deliver, and/or support the intervention should be involved.

This might include

- *People living with dementia and/or unpaid carers*
- *Health or social care professionals*
- *Healthcare managers, insurance companies, or policy makers*
- *Other people relevant to the intervention*

* must provide value

- No, not particularly important
- Yes, important
- Yes, very important
- Unable to provide feedback
- Prefer not to answer

(4) When developing, testing, evaluating, and/or implementing a psychosocial intervention,

changes to the intervention should be made, when necessary.

Changes might include adaptations of the content, format, delivery, or other intervention aspects.

* must provide value

- No, not particularly important
- Yes, important
- Yes, very important
- Unable to provide feedback
- Prefer not to answer

(5) When developing, testing, evaluating, and/or implementing a psychosocial intervention,

how much the intervention costs and who will pay for it should be considered**.**

* must provide value

- No, not particularly important

- ◯ Yes, important
- ◯ Yes, very important
- ◯ Unable to provide feedback
- ◯ Prefer not to answer

(6) When developing, testing, evaluating, and/or implementing a psychosocial intervention,

anything related to the intervention that is unclear should be identified.

Things that are unclear might be related to the research itself, such as how to recruit participants, or the delivery of the intervention, such as which skills and knowledge health and social care professionals need to deliver it, or preferences of people with dementia and/or carers.

* must provide value

- ◯ No, not particularly important
- ◯ Yes, important
- ◯ Yes, very important
- ◯ Unable to provide feedback
- ◯ Prefer not to answer

What else is important for researchers to consider when developing, testing, evaluating, and/or implementing a psychosocial intervention for people with dementia and/or unpaid carers?

To submit your responses, please **click on the 'submit survey' button below.**

If you have any queries or complaints about this study, you can contact:

Dr Sara Laureen Bartels, researcher at Maastricht University,

sara.bartels@maastrichtuniversity.nl, phone +31 622 875 918

Dr Federica D'Andrea, researcher at University of West London,

federica.dandrea@uwl.ac.uk, phone: +44 208 209 4208

Submit

Save & Return Later

Powered by REDCap - Cookie policy

Survey Queue

AAA

Survey for people with lived experience of dementia

Thank you for signing informed consent!

Please state the main survey by **clicking the button 'BEGIN SURVEY' below**.

Please complete the following demographic questions.

Age

Sex

* must provide value

- ◯ Born Female
- ◯ Born Male
- ◯ Prefer not to answer

Gender

* must provide value

- ◯ Woman
- ◯ Man
- ◯ Self-describe
- ◯ Prefer not to answer

Country of residence

We are interested in your opinion on non-pharmacological support, also known as '**psychosocial interventions**', for people living with dementia and/or unpaid carers, which may include, for example:

- Talk therapy to support coping with challenges in everyday life
- Speech and language therapy to help with speaking and understanding others
- Sensory stimulation, like massages or touch therapy
- Exercise, such as walking or yoga
- Group activities to feel connected and understood, including dementia cafés and support groups
- Education to know what dementia is and how to live with it
- Cognitive therapy to improve memory
- Environmental changes such as the use of lights, objects, or safe layout to help the person feel comfortable in their space

These interventions all aim to improve quality of life and **do not involve taking medication**. Quality of life could include the person's well-being, daily functioning, involvement in activity, mood, relationships, or any

other aspect of their life that is relevant to them.

In this study, we want to understand **what is important** when:

1. Developing, meaning creating a psychosocial intervention
2. Testing, meaning checking if a psychosocial intervention is easy to use, acceptable, practical, and feasible
3. Evaluating, meaning examining a psychosocial intervention and checking it if works
4. Implementing, meaning putting a psychosocial intervention into real-life and everyday settings

Please answer the following questions:

Which, if any, psychosocial intervention(s) have you previously used or are currently using?

* must provide value

- [ ] Talk therapy/ psychotherapy to support coping with challenges in everyday life
- [ ] Speech and language therapy to help with speaking and understanding others
- [ ] Sensory stimulation, like massages or touch therapy
- [ ] Exercise/ physical activity, such as walking or yoga
- [ ] Group activities to feel connected and understood, including dementia cafés and support groups
- [ ] Education to know what dementia is and how to live with it
- [ ] Cognitive therapy or rehabilitation to improve memory
- [ ] Environmental changes such as the use of lights, objects, or safe layout to help the person feel comfortable in their space
- [ ] Occupational therapy to help with daily activities
- [ ] Learning a new skill
- [ ] Other
- [ ] None
- [ ] Prefer not to answer

If you selected 'other', please add details here:

Have you previously been **involved or participated in a psychosocial intervention research project**?

Specifically, a project focused on a psychosocial intervention for people living with dementia and/or unpaid carers

* must provide value

- [ ] No, I have never participated in an intervention research project
- [ ] Yes, the intervention was being develop or (co-)created
- [ ] Yes, the intervention was being tested to see it works
- [ ] Yes, the intervention was evaluated, to check if or how it works, or not
- [ ] Yes, the intervention was being put into real life practice (implementation)
- [ ] I don't know
- [ ] Prefer not to answer

Below, you will see **six statements** of what might be important when **developing, testing, evaluating, and/or implementing psychosocial interventions for people living with dementia and/or unpaid carers.**

Please indicate how important you think these aspects are.

(1) When developing, testing, evaluating, and/or implementing a psychosocial intervention,

it should be consider **where the intervention will be used.**

Interventions might be used at home, at day care, at a hospital, in public, online, or other places.

* must provide value

- ◯ No, not particularly important
- ◯ Yes, important
- ◯ Yes, very important
- ◯ Unable to provide feedback
- ◯ Prefer not to answer

(2) When developing, testing, evaluating, and/or implementing a psychosocial intervention,

the **reason why and how an intervention might add value** should be explored.

This reason might be based on a theoretical explanation or hypothesis, previous research, or expressed needs.

* must provide value

- ◯ No, not particularly important
- ◯ Yes, important
- ◯ Yes, very important
- ◯ Unable to provide feedback
- ◯ Prefer not to answer

(3) When developing, testing, evaluating, and/or implementing a psychosocial intervention,

the **people who will receive, deliver, and/or support the intervention should be involved.**

This might include

- *People living with dementia and/or unpaid carers*
- *Health or social care professionals*
- *Healthcare managers, insurance companies, or policy makers*
- *Other people relevant to the intervention*

* must provide value

- ◯ No, not particularly important
- ◯ Yes, important
- ◯ Yes, very important
- ◯ Unable to provide feedback

◯ Prefer not to answer

(4) When developing, testing, evaluating, and/or implementing a psychosocial intervention,

changes to the intervention should be made, when necessary.

Changes might include adaptations of the content, format, delivery, or other intervention aspects.

* must provide value

◯ No, not particularly important
◯ Yes, important
◯ Yes, very important
◯ Unable to provide feedback
◯ Prefer not to answer

(5) When developing, testing, evaluating, and/or implementing a psychosocial intervention,

how much the intervention costs and who will pay for it should be considered**.**

◯ No, not particularly important
◯ Yes, important
◯ Yes, very important
◯ Unable to provide feedback
◯ Prefer not to answer

(6) When developing, testing, evaluating, and/or implementing a psychosocial intervention,

anything related to the intervention that is unclear should be identified.

Things that are unclear might be related to the research itself, such as how to recruit participants, or the delivery of the intervention, such as which skills and knowledge health and social care professionals need to deliver it, or preferences of people living with dementia and/or carers.

* must provide value

◯ No, not particularly important
◯ Yes, important
◯ Yes, very important
◯ Unable to provide feedback
◯ Prefer not to answer

What else is important for researchers to consider when developing, testing, evaluating, and/or implementing a psychosocial intervention for people living with dementia and/or unpaid carers?

I wish to receive financial reimbursement (£12.50/15euro) for completing this survey.

* must provide value

◯ Yes

◯ No

To submit your responses, please **click on the 'submit survey' button below.**

If you have any queries or complaints about this study, you can contact:

Dr Sara Laureen Bartels, researcher at Maastricht University,

sara.bartels@maastrichtuniversity.nl, phone +31 622 875 918

Dr Federica D'Andrea, researcher at University of West London,

federica.dandrea@uwl.ac.uk, phone: +44 208 209 4208

Submit

Save & Return Later

Powered by REDCap - Cookie policy
