# Supplementary material for: Which methodologies and methods should be used for psychosocial intervention research in dementia? Protocol for a stakeholder-driven, multi-method Delphi study
Source: BMJ Open. 2026 Sep 2;16(9):e122720. doi: 10.1136/bmjopen-2026-122720 (PMC13548282; doi:10.1136/bmjopen-2026-122720)
Supplement: Supplementary file 3 [file bmjopen-2026-122720.reviewer_comments.pdf]

PEER REVIEW HISTORY

BMJ Open publishes all reviews undertaken for accepted manuscripts. Reviewers are asked to complete a checklist review form and are provided with free text boxes to elaborate on their assessment. These free text comments are reproduced below.

ARTICLE DETAILS

Title (Provisional)

Which methodologies and methods should be used for psychosocial intervention research in dementia? Protocol for a stakeholder-driven, multi-method Delphi study

Authors

Bartels, Sara Laureen; D'Andrea, Federica; Burnand, Alice; De Bruin, Simone R; Chirico, Ilaria; Elliott, Emma; Flynn, Aisling; Garcia, Lesley; Gebhard, Doris; Handley, Melanie; Janssen, Niels; Masterson-Algar, Patricia; Markaryan, Marine; Roes, Martina; Stephens, Nathan; Van den Block , Lieve; Moniz-Cook , Esme; Graff, Maud

---

VERSION 1 - REVIEW

**Reviewer**	**1**
**Name**	**Pond, Dimity**
**Affiliation**	**University of Newcastle Australia, General Practice**
**Date**	**25-Jul-2026**
**COI**	**None**

This is an interesting and well written protocol on an important topic: strengthening the evidence base for core elements, methodologies and methods for psychosocial interventions in dementia.

I have some comments:

*I note on page 10 reference to an ongoing scoping review on methods and methodologies that will feed into Phase 3. How will the authors cope with new additions and insights emerging from the scoping review in parallel with their work?

*Minor formatting issues:

-Page 12 states that the design of the study is illustrated in figure 2. However figure 2 is a sample survey and I believe figure 1 is the study design.

-There are some difficulties around past and present test. The authors transparently state that they have completed some components of the study. However it is awkward to read past and present tense within a single sentence, eg, 'one researcher created codes and themes and selects quotations' p21 under 2.4.3. I believe this would be better avoided.

VERSION 1 - AUTHOR RESPONSE

Reviewer: 1
Dr. Dimity Pond, University of Newcastle Australia
Comments to the Authors:
This is an interesting and well written protocol on an important topic: strengthening the evidence base for core elements, methodologies and methods for psychosocial interventions in dementia.

>>> Response from authors: Thank you, Dr Dimity Pond, for taking the time to review our protocol and for your kind words. By addressing your comments and revising the manuscript, we hope to have improved the quality.

I have some comments:
*I note on page 10 reference to an ongoing scoping review on methods and methodologies that will feed into Phase 3. How will the authors cope with new additions and insights emerging from the scoping review in parallel with their work?

>>> Response from authors: While the scoping review is being conducted in parallel to the Delphi study, data extraction is already resulting in a preliminary overview of methodologies and methods used to develop, test, evaluate, and implement psychosocial interventions for dementia. Specifically, this overview will be compared with those mentioned in Phase 1 and added to the consensus items in Phase 3. If additional survey rounds should be required, the final overview from this review will also be used. This information was added to section 2.5 and 2.6.

*Minor formatting issues:

-Page 12 states that the design of the study is illustrated in figure 2. However figure 2 is a sample survey and I believe figure 1 is the study design.

>>> Response from authors: This was an error, and it was corrected to refer to Figure 1.

-There are some difficulties around past and present test. The authors transparently state that they have completed some components of the study. However it is awkward to read past and present tense within a single sentence, eg, 'one researcher created

codes and themes and selects quotations' p21 under 2.4.3. I believe this would be better avoided.

>>> Response from authors: We agree, the differences in tenses impacts the readability. We adjusted the text to future tense where possible, and only used past tense when an action is completed, and present tense, if the statement is generally valid.

*** ***

COI statement(s):

Reviewer: 1
If you have selected 'Yes' above, please provide details of any competing interests.: Not applicable.
